# Biomedical Applications of Electrets: Recent Advance and Future Perspectives

**DOI:** 10.3390/jfb14060320

**Published:** 2023-06-12

**Authors:** Xinyuan Zhang, Jiulong Zhao, Pei Xie, Shige Wang

**Affiliations:** 1 School of Health Science and Engineering, University of Shanghai for Science and Technology, No. 516 Jungong Road, Shanghai 200093, China; zxy_work1997@163.com; 2 Department of Gastroenterology, Changhai Hospital, Naval Medical University, No. 168 Changhai Road, Shanghai 200433, China; jlzhao9@163.com (J.Z.); winner0119@sina.com (P.X.); 3 School of Materials and Chemistry, University of Shanghai for Science and Technology, No. 516 Jungong Road, Shanghai 200093, China

**Keywords:** electret, inorganics, biomacromolecules, biomedical application

## Abstract

Recently, electrical stimulation, as a non-pharmacological physical stimulus, has been widely exploited in biomedical and clinical applications due to its ability to significantly enhance cell proliferation and differentiation. As a kind of dielectric material with permanent polarization characteristics, electrets have demonstrated tremendous potential in this field owing to their merits of low cost, stable performance, and excellent biocompatibility. This review provides a comprehensive summary of the recent advances in electrets and their biomedical applications. We first provide a brief introduction to the development of electrets, as well as typical materials and fabrication methods. Subsequently, we systematically describe the recent advances of electrets in biomedical applications, including bone regeneration, wound healing, nerve regeneration, drug delivery, and wearable electronics. Finally, the present challenges and opportunities have also been discussed in this emerging field. This review is anticipated to provide state-of-the-art insights on the electrical stimulation-related applications of electrets.

## 1. Introduction

In recent years, physiologically relevant electrical stimulation has drawn lots of attention in the fields of tissue engineering and biomedicine by virtue of the characteristics of non-pharmacological physical stimulation [1,2,3]. It could enhance cell proliferation and differentiation, improve cellular behavior, and affect the intracellular microenvironment [4,5,6]. Due to these effects, electrical stimulation shows great potential in a wide range of applications, including wound healing, tissue engineering, neural stimulation, and bone regeneration [7,8]. Nevertheless, the side effects of excessive electrical stimulation might be detrimental, such as rhabdomyolysis and acute myopathy [9], and therefore the selection of safer electroactive materials and parameters will appear to be particularly important and relevant.

Electret, as a promising electroactive agent characterized by permanent polarization, can provide endogenous electrical stimulation after being subjected to an external electric field [10,11,12,13]. The word “electret” was first coined in 1892 by Oliver Heaviside to describe dielectric materials with permanent polarization [14,15]. However, the history of electrets can be traced back to the year 1732. At that time, a British scientist named Stephen Gray discovered that waxes and resins can attract light bodies permanently, which became known as “perpetual attractive power” [16]. A century later, the British scientist Michael Faraday began working on electrets and came up with a theoretical explanation of “perpetual attractive power” [14]. The first electret was made by the Japanese physicist Mototarô Eguchi from carnauba wax (material from the Brazilian palm), which has displayed a strong external electric field till now [16,17,18,19]. Bernhard Gross, another pioneer in electret research, investigated carnauba wax electrets and proposed hypotheses about the origin of the permanent polarization of electrets as two continuous processes (orientation of molecular dipoles and space charges and injection of charges from electrodes) [20]. Since then, electrets have become the focus of research and have been systematically investigated in diverse fields.

An electret can be polarized by an external electric field and charged [21,22]. These charges, which can be dipole charges and space charges (e.g., surface charges and bulk charges), are stored on the surface of the electret or throughout the space of the electret [16,23]. The dipole charge is formed by orienting the electrets under an electric field at high temperatures, whereas the space charge can be obtained by injecting charge carriers directly into the electret [24]. As a consequence, the performance of electrets is usually evaluated by charge density (the density of electret space charge or polarization charge, including surface charge density and space charge density) and charge storage lifetime (interpreted as the effective decay time constant of the electret charge) [25]. An effective electret can retain charge semi-permanently or permanently after the removal of the electric field and is not susceptible to external stimuli. In addition to charge density and charge storage lifespan, the composition of dielectric agents is also a key factor affecting the performance of the electret [26,27]. Typical representatives of electret are shown in Table 1. Silicon dioxide (SiO_2_) [28,29], aluminum oxide (Al_2_O_3_) [30,31], zinc oxide (ZnO) [32,33], and hydroxyapatite (HA) [34,35] are widely used inorganic electret materials, which have the advantage of excellent charge retention ability and high surface charge density. More importantly, good biocompatibility supports their further potential applications in biomedicine [10,14]. In addition, different categories of biomacromolecules, such as polyethylene (PE) [36,37], polypropylene (PP) [38,39], polytetrafluoroethylene (PTFE) [40,41], fluorinated ethylene propylene (FEP) [42,43], polyimide (PI) [44,45], chitosan (CS) [46,47] and poly(ethylene terephthalate) (PET) [48,49], also exhibit electret properties. These polymers are either highly insulating non-polar polymers (high insulation ensures good charge storage properties) or strongly polar polymers with dipole moments [14,23]. Furthermore, they are biologically active or inert to varying degrees and have been used for the repair and replacement of sclerotic or soft tissues [50,51,52]. It is worth noting that, several reviews of electret have been published in recent years. These articles summarize the application of electrets in air filtration, energy harvesting, and energy storage. However, there is a lack of comprehensive reviews of the biomedical applications of electrets in biomedicine.

**Figure 1:**
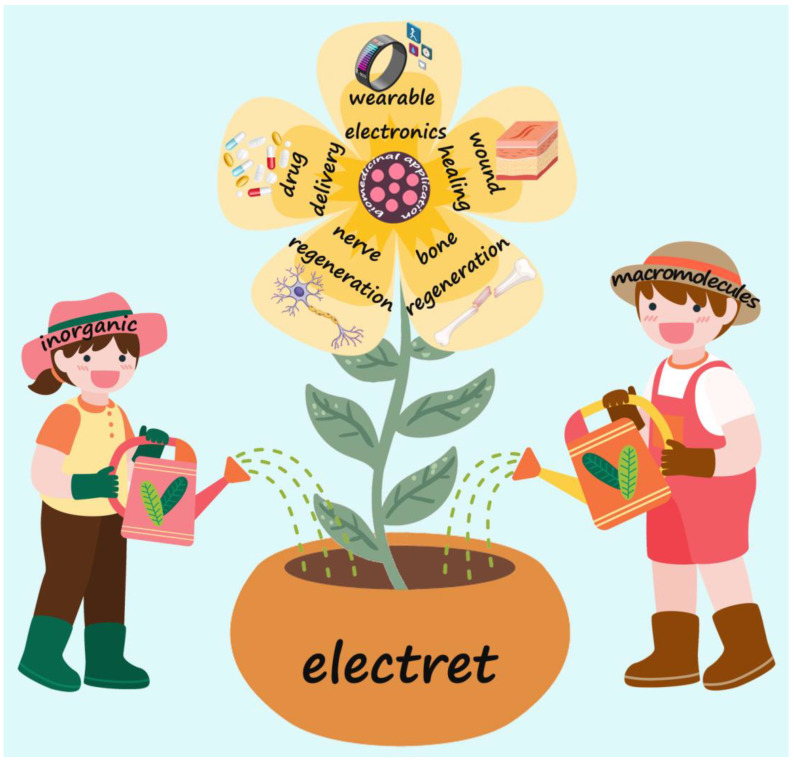
Schematic representation of the classification of electrets and their biomedical applications.

In this review, we provide an overview of the design, preparation, and biomedical applications of inorganic and macromolecule electrets (Figure 1). In the first part of the review, we briefly introduce the electret and its discovery and development processes. Then, in the second part, the main categories, fabrication methods, and primary features of electrets are summarized and discussed in detail. The third part focuses on the applications of electrets in bone regeneration, wound healing, nerve regeneration, drug delivery, and wearable electronics. Finally, in the fourth part, some perspectives and challenges associated with the future development of electrets are also discussed.

## 2. Classification and Fabrication

### 2.1. Inorganics

#### 2.1.1. Silicon Dioxide (SiO_2_)

SiO_2_, an acid oxide abundant in nature, is one of the most important compounds of silicon. It has been widely used as an electret material because of its excellent charge storage capacity and good biocompatibility [10,64]. Minami et al. prepared SiO_2_ thin film electrets using radio frequency (RF) magnetron sputtering [65]. In this research, SiO_2_ films were prepared on the substrate of the silicon wafer by using a fused silica target. Then, sputter deposition was performed at a substrate temperature of about 360 °C, a sputtering gas pressure (Ar + O_2_) of 0.35 Pa, and an O_2_ partial pressure of 20%. After deposition, the SiO_2_ film is placed on a mobile platform and charged with air at room temperature using a corona discharge method. The electret exhibited high surface potential stability in a highly humid environment, where the voltage was maintained at about 300 V.

**Figure 2:**
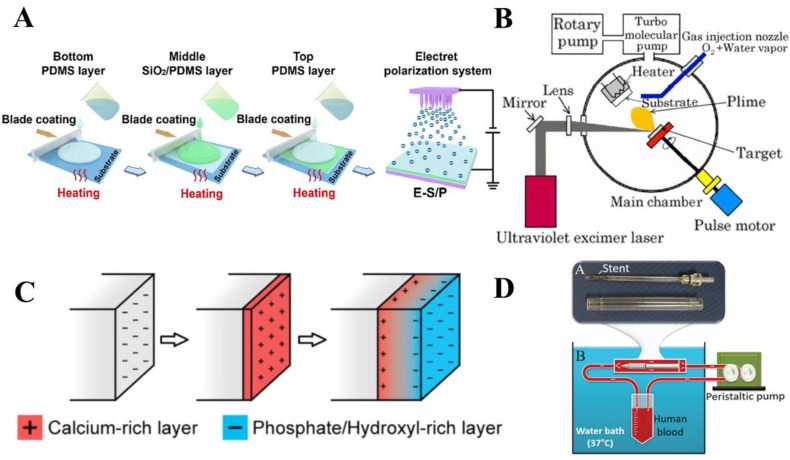
Schematic diagram of typical inorganic electrets and their preparation. (**A**) Schematic diagram of the preparation of SiO_2_/PDMS composite film. A PDMS film containing SiO_2_ electret was prepared by layer-by-layer coating and polarizing. Reprinted with permission from Ref. [10]. Copyright 2022, copyright American Chemical Society. (**B**) Schematic diagram of HA electret films prepared by the PLD method. Reprinted with permission from Ref. [70]. Copyright 2013, copyright Hilaris. (**C**) The process of electrical polarization in HA during electrochemical synthesis. Reprinted with permission from Ref. [71]. Copyright 2015, copyright American Chemical Society. (**D**) Annular perfusion model of Ta_2_O_5_ electret coating. Reprinted with permission from Ref. [55]. Copyright 2022, copyright AIP Publishing.

In another study, Qiao et al. designed the SiO_2_ electret-polydimethylsiloxane (SiO_2_/PDMS) composite electroactive films to provide stable and long-lasting endogenous electrical stimulation and induce effective bone regeneration [10]. SiO_2_ electrets were uniformly dispersed in the PDMS matrix, and the sandwich composite film was prepared by a simple layer-by-layer coating method (Figure 2A). Firstly, SiO_2_ nanoparticles were dispersed in hexamethylene by ultrasound to obtain uniform SiO_2_ dispersions. The SiO_2_/PDMS composites were prepared by adding SiO_2_ dispersion to PDMS. The degassed PDMS precursor was scraped onto the substrate while cured at 60 °C to form the bottom PDMS layer. Then, the SiO_2_/PDMS composites were scraped and solidified at 60 °C on the prepared PDMS layer. Another PDMS precursor was cross-linked by scraping at 60 °C to obtain the upper PDMS layer and form three complete sandwich-like films. After encapsulation, electret polarization was carried out to obtain the composite electroactive films. By adjusting the electret concentration, the composite film showed a stable Zeta potential (−61.5 mV) over an observation period of up to 42 days, which matched the range of natural biological potentials. The result of in vitro biological experiments showed that SiO_2_/PDMS films could effectively promote cell activity and enhance the osteogenic differentiation of bone marrow mesenchymal stem cells (BMMSCs).

#### 2.1.2. Hydroxyapatite (HA)

HA, Ca_10_(PO_4_)_6_(OH)_2_, a calcium phosphate crystal with monoclinic or hexagonal crystal symmetry, is a major component of hard tissues in humans and animals [66,67]. As a proton conductor, HA can be polarized under an external electric field at high temperatures and maintain this polarized state [66,68]. The polarized surface of HA is able to combine with living bone tissue and exhibits good bioelectrical activity, osteoconductivity, and biocompatibility [69]. To measure the piezoelectric properties of HA films, Nishigaki et al. used the Pulsed Laser Deposition (PLD) method to deposit HA films on Ti or Pt substrates [70]. A schematic diagram of the device is shown in Figure 2B. Firstly, HA particles were placed in a vacuum chamber. Then, molecules, atoms, and ions on the target surface were irradiated by a KrF excimer laser beam in a vacuum chamber and reacted with atmospheric pressure on a Ti or Pt substrate. The energy density of the laser on the target is about 1 J/cm^2^, and the pulse repetition rate is 10 Hz. The film deposition rate is about 10 nm/min and the total thickness is 1.5 µm. The piezoelectric properties of the HA film were finally estimated by measuring the vibration response of mechanical strain and voltage simultaneously, and the piezoelectric constant d_31_ was estimated to be about 0.1 pC/N.

In a subsequent study, Fu et al. reported a polarization mechanism for the electrochemical synthesis of HA film [71]. HA film was synthesized on the surface of Ti and stainless-steel electrodes. The concentration of ionic reactants in the electric double layer of the HA film surface is affected by the driving potential, as illustrated in Figure 2C. Due to the compositional gradient induced in the HA film, a large polarization is formed in the direction perpendicular to the film surface. The HA film synthesized by electrochemistry has a high stored charge of 70,000 μC/cm^2^. This novel polarization mechanism in the synthesis process provides a larger stored charge than the post-synthetic polarization of ferroelectric or electret materials.

#### 2.1.3. Tantalum Pentoxide (Ta_2_O_5_)

Ta_2_O_5_ is an important transition metal oxide with high mechanical strength, good thermal stability, and good biocompatibility [55]. As a dielectric material, Ta_2_O_5_ has a high permittivity and low dielectric loss, and it is easy to obtain the electret state with a negative charge [55,72,73]. Zafar et al. proposed a new electret coating technology that can generate a lasting negative charge on the surface of the stent to make the stent have an anti-thrombotic nature, as shown in Figure 2D [55]. Before coating, the surface of the support was thoroughly cleaned. Then, the stent was vacuum sprayed using a vacuum plasma spray technique, where Ta_2_O_5_ molecules and ions were sprayed onto the stent surface at a high speed. A ring perfusion model under confocal microscopy was used to assess stent thrombogenicity and determine the effectiveness of electret coating techniques in conferring antithrombotic properties on standard stents. The experimental results demonstrated that the electret coating imparted significant antithrombotic properties to the stent. Moreover, the Ta_2_O_5_ coating is light and opaque and does not require additional contrast agents, making the fabrication of the stent more cost-effective.

#### 2.1.4. Titanium Dioxide (TiO_2_)

TiO_2_ is an efficient, stable, and green photocatalytic material with a variety of crystal structures that has long-term stability, chemical inertness, corrosion resistance, and non-toxicity characteristics [74,75]. With good charge storage capacity, TiO_2_ has been regarded as a suitable material for dielectrics and transparent conductors [76]. Bandyopadhyay et al. changed the surface of commercially pure Ti by growing TiO_2_ via anodizing, and then introduced surface charges to form bio-electrets by electrothermal polarization [77]. TiO_2_ nanotubes were manufactured with CpTi wafers in a glycol-based electrolyte containing 1% HF with a potential difference of 40 V. The sample was rinsed thoroughly in deionized water to wash off residual HF. Subsequently, they were dried at 40 °C. Finally, the sample was heated to 300 °C and then polarized with a DC field of 2 kV/cm for 1 h. The result showed that the stored charge on the TiO_2_ surface was 37.15 ± 14 mC/cm^2^, and the samples did not have any charge leakage for 18 months.

### 2.2. Macromolecules

#### 2.2.1. Polyethylene (PE)

Because of its low cost, easy processability, and environmental friendliness, PE has become a good choice for the preparation of different materials [78]. In addition, it also has the advantages of excellent ductility, high hardness, corrosion resistance, and outstanding electrical insulation. Furthermore, the excellent long-term durability and biocompatibility of PE have received lots of attention [37,79]. For these reasons, PE has been invited as a base material in the manufacturing of electrets. Earlier studies have confirmed that the electret properties of PE can be improved by introducing different additives into PE. For example, Galikhanov et al. studied the influence of montmorillonite on the properties of PE [80]. The mixture of PE and montmorillonite was prepared with a mixer at 180 °C for 5 min. The polymer composite was heated to 190 °C, pressurized for 3 min, and cooled under pressure. Finally, a film with a thickness of 200 μm was obtained. The polymer film was charged in a corona discharge field generated by an electrode consisting of 196 needles evenly distributed over a square of 49 cm^2^. Before polarization, the film was heated to 100 °C and then polarized for 30 s by applying a corona discharge voltage of 30 kV. The results proved the enhancement of electret characteristics and the improvement of stability.

**Figure 3:**
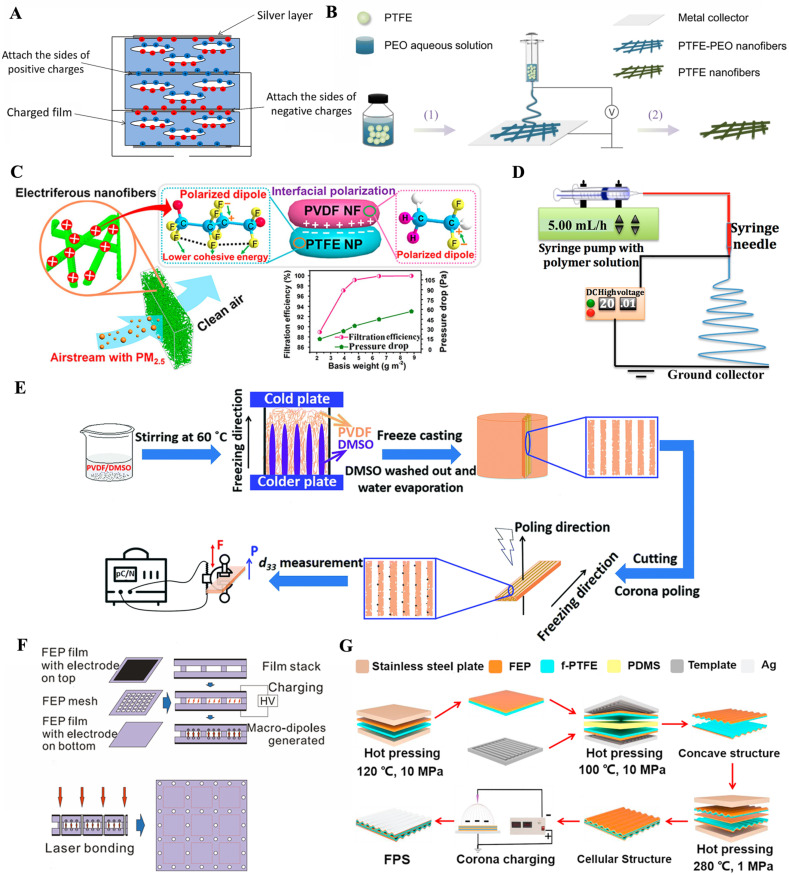
Schematic diagram of typical biomacromolecule electrets and their preparation. (**A**) Schematic of a multilayer iron-electret PE system. Reprinted with permission from Ref. [57]. Copyright 2019, copyright John Wiley and Sons, Inc. (**B**) Schematic diagram of PTFE nanofiber film preparation: (1) PTFE-PEO nanofiber film was prepared by electrospinning, and (2) PEO was removed from the electrospun PTFE-PEO nanofiber film by heat treatment. Reprinted with permission from Ref. [86]. Copyright 2019, copyright Springer Nature. (**C**) Schematic description of nanofiber film preparation (PVDF as matrix polymer and PTFE nanoparticles as excitation charge enhancer). Reprinted with permission from Ref. [87]. Copyright 2016, copyright American Chemical Society. (**D**) Schematic of preparation of PVDF fiber mat using a syringe pump electrospinning device with a rotary collector. Reprinted with permission from Ref. [90]. Copyright 2016, copyright Multidisciplinary Digital Publishing Institute. (**E**) Schematic diagram of the process of obtaining porous polymers by freeze casting (P is the direction of polarization and F is the direction of force). Reprinted with permission from Ref. [60]. Copyright 2018, copyright Royal Society of Chemistry. (**F**) Preparation of a three-layer electret: A laser-cut polymer grid is sandwiched between two uniform polymer films and charged with a high DC voltage. After that, the laser beam is used for local fusion at selected points on the grid fringe. Reprinted with permission from Ref. [92]. Copyright 2010, copyright Springer Nature. (**G**) The design diagram of FPS cellular structure by hot pressing method and corona charging with a high voltage source. Reprinted with permission from Ref. [93]. Copyright 2017, copyright Elsevier.

Moreover, PE may facilitate the formation of electrets with cellular structure, which are usually produced by introducing a foaming agent into a polymer matrix. The foamed polymer not only makes it a lightweight electret but also improves its electrical properties [57,81]. As shown in Figure 3A, Hamdi et al. prepared electrets with excellent piezoelectric coefficients using PE as a raw material by the “extrusion film blowing continuous chemical foaming method” [57]. The films were further treated via the thermal pressure method by raising the temperature at a constant speed from 25 to 81 °C and maintaining it for 14 min to achieve stability. Then, a constant pressure was maintained during the slow cooling of the sample until ambient temperature to preserve the cellular structure. Finally, the piezoelectric properties of the film were obtained through a corona charging process. The initial d_33_ of the piezoelectric film was 1315 pC/N, and the stable value was 792 pC/N after 50 days. This study represents a new step towards the goal of improving the piezoelectric properties of cellular PE films.

#### 2.2.2. Polypropylene (PP)

As a nonpolar polymer, PP is able to exhibit good piezoelectric properties after a proper charge polarization, and it has become one of the most widely used electrets due to its good flexibility and large d_33_ coefficient [14,82]. However, the disadvantage of low surface-charge stability often limits its application [23]. Therefore, efforts have been made to improve the electrical performance of PP. Mohebbi et al. prepared PP foam film by supercritical N_2_ continuous extrusion and charged it using a corona discharging method [83]. The physical foaming was carried out by continuous extrusion on the co-rotating twin screw extruder. In order to control the morphology, the sample was drawn uniaxially in the melt state. To get optimal piezoelectric properties, the screw structure, temperature profile, stretching speed, nucleating agent, and other process parameters and components were optimized. The results showed that the optimal piezoelectric d_33_ coefficient is 550 pC/N when N_2_ is used as an ionized gas; however, the maximum value is only 250 pC/N when using air.

In addition, the optimal piezoelectric property was related to the surface morphology of files. Wang et al. demonstrated that the surface treatment of PP-film with H_3_PO_4_ could improve its charge stability [84]. In their attempts, one side of a PP film with a thickness of 50 µm was first plated with 10 nm of Cr, followed by 100 nm of Al, allowing for good electrical contact during charging and charge attenuation measurements. Three sets of PP samples were used in the experiment: original PP (not processed), thermally treated PP (annealing at 120 °C for 24 h in the absence of H_3_PO_4_), and chemically treated PP (covering the nonmetallic surface with H_3_PO_4_ and treating it at 120 °C for 24 h), and the surface was characterized by SEM. The experimental results showed that the modification of H_3_PO_4_ increased the thermal stability of positively charged PP and negatively charged PP. Moreover, the location of the surface trap distribution also moved from 1.05 eV to 1.16 eV and 1.19 eV, respectively.

#### 2.2.3. Polytetrafluoroethylene (PTFE)

Due to the helical chain structure being uniformly covered by F atoms on the carbon skeleton and the effective shielding effect of F atoms on the positive charge of C atoms on the main chain, PTFE has good flexibility, a low friction coefficient, a low dielectric constant, a high chemical inertia, and a high heat resistance [40,41,85]. Therefore, PTFE has been considered a good choice as an electrostatic dielectric material. In a study, Lin et al. reported a high-performance electret of electrospun PTFE nanofiber film [86]. As shown in Figure 3B, the PTFE nanofibrous film was prepared through a two-step process. Firstly, PTFE-PEO (polyethylene oxide) nanofiber film was prepared through electrospinning by adding PTFE and PEO to deionized water to form a uniform suspension. Firstly, the mixture solution in the syringe was pumped from the tip by applying a high voltage of 25 kV. Subsequently, the prepared PTFE-PEO nanofiber film was heated at 350 °C for 10 min to remove the PEO component and obtain the PTFE nanofiber film. The tensile strength of the prepared PTFE nanofiber film is 3.8 MPa and the surface potential is stable at −270 V, showing its excellent mechanical properties. Meanwhile, it has a peak power of 56.25 µW and long-term cycle stability, which shows its potential for applications in sensitive self-powered wearable devices.

Another approach to improving the surface potential and enhancing the charge stability was proposed by Wang et al. [87]. As depicted in Figure 3C, PVDF was used as the matrix polymer, and PTFE nanoparticles (PTFE NPs) were used as the excitation charge enhancer. The PVDF/PTFE NPs polymer solution was aspirated into a syringe fixed to a supporting frame. Then, a uniform PVDF/PTFE solution was extruded through a 5 G metal needle at a controllable injection rate, and a stable jet was formed by simultaneously applying a DC voltage of 30 kV at the precise location of the needle. Finally, the prepared PVDF/PTFE fiber film was assembled on a ground metal roller barrel covered by a nonwoven substrate and rotated at 50 rpm. The results showed that the fiber film has good charge stability with long-term duration and filtration efficiency of up to 99.972% with a low-pressure drop of 57 Pa and a quality factor of 0.14 Pa^−1^.

#### 2.2.4. Polyvinylidene Fluoride (PVDF)

PVDF is a semi-crystalline and non-reactive thermoplastic polymer popular for its outstanding piezoelectric properties, thermal stability, and chemical resistance [88,89]. Because of its unique molecular structure, PDVF is able to maintain a static charge for a long time [90]. In addition, PVDF is capable of generating electrical signals in response to sound, force, pressure, or heat and has thus been frequently applied as electrets. Lolla et al. prepared PVDF fiber mats by a controlled electrospinning process to improve the electrostatic properties of PDVF [90]. Figure 3D shows the schematic of the electrospinning apparatus, where a 5 mL plastic syringe filled with a solution was heated to 70 °C and attached to a stainless-steel needle with a Teflon tube. A fiber pad with an average specific gravity of 20 g/m^2^ was obtained using the vertical electrospinning method. Finally, the resulting electrospun sheet was heated at 70 °C for 2 h to allow the residual solvent to evaporate. The performance of the electret was tested iteratively to observe the charge loss as a function of time, which showed that the PVDF fiber mats still maintained the static charge and the trapping properties over 11 months.

In a subsequent study, Zhang et al. developed a new cost-effective and environmentally friendly strategy for the preparation of porous PVDF electret materials by the freezing casting method [60]. As shown in Figure 3E, the prepared PVDF/DMSO (dimethyl sulfoxide) solution was poured into a PDMS mold with the bottom surface placed in liquid N_2_, and then the top surface of the mold was placed at 20 °C for unidirectional freezing. After freezing, the samples were de-molded and immediately immersed in water to remove the solidified DMSO. The samples were then dried at 40 °C for 24 h to remove the remaining moisture. The dried samples were frozen in liquid N_2_ and then cut parallel to the freeze-casting direction to a thickness of 1.5 mm. A DC voltage of 26.8 kV was then used for corona polarization. The results showed that the long-range arrangement of the channels during directional freezing is conducive to the formation of highly polarized structures with a high d_33_ (264 pC/N). This new approach opens the way for creating tailor-made pore structures and voids in electret materials.

#### 2.2.5. Fluorinated Ethylene Propylene (FEP)

FEP is famous for its excellent biological inertness and thermal charge stability, as well as being hydrophobic and non-stick to most materials, and because of this, it has shown outstanding performance as an electret [11,91]. Fang et al. used FEP copolymer films to fabricate three-layer piezoelectrets by laser cutting, laser bonding, electrode evaporation, and high-field polarization [92]. As illustrated in Figure 3F, an FEP grid was sandwiched between two homogeneous FEP films whose outer surfaces were metalized with 50 nm thick Al electrodes. A grid of regular square holes (3 × 3 mm^2^) was prepared by laser cutting and separated by FEP stripes (1 mm wide). Then, internal polarization was performed by applying a DC voltage to the electrode. After polarization, a 0.1 mm diameter laser beam was applied to the selected point on the grid fringe to melt and fuse the film. After electric charging, the three-layer piezoelectret exhibited a highly piezoelectric d_33_ coefficient of approximately 350 pC/N, which had only a slight decay at 120 °C, and still maintained about 70% of that even at 130 °C.

Another method of creating micron-sized voids was developed by Wang et al. [93]. In this study, an ultra-sensitive cellular fluorocarbon piezoelectret pressure sensor (FPS) was fabricated by a simple three-step hot-pressing process (Figure 3G). In the first step, the dense FEP and the fibrous PTFE (f-PTFE) with thicknesses of 50 µm and 25 µm, respectively, were stacked between two stainless steel plates with an area of 5 × 5 cm^2^, and the bilayer structure of the FEP/f-PTFE composite polymer was obtained by hot pressing at 120 °C and 10 MPa for 3 min. Secondly, the composite polymer, with PDMS rubber as a buffer layer, was sandwiched between two pattern-based metal templates and hot-pressed at 100 °C for 3 min (10 MPa). In the third step, the sandwiched FEP/f-PTFE film, FEP film, and FEP/f-PTFE film were applied with a small pressure (1 MPa) and a high temperature (280 °C) and extruded for 5 min to obtain the cellular structure of the composite fluorocarbon polymer. Subsequently, a negative corona charge was used to generate a macroscopic dipole inside the cellular fluorocarbon polymer. The sample was placed below the corona needle, and a high-voltage of −15 kV was applied for 3 min. Finally, by RF magnetron sputtering, the silver electrode was metalized on both sides of the film, and a uniform FPS was obtained. The FPS exhibited significant pressure sensitivity up to 7380 pC/N in the delicate pressure regime (less than 1 kPa) and high stability.

#### 2.2.6. Chitosan (CS)

Chitosan is a water-soluble polysaccharide that is abundant in nature [94,95]. As a biological electret, the electret state of CS is mainly attributed to its high polarization storage induced by the -OH group and bound water, and the electric charge can be maintained for a long time after the removal of the electric field. CS has outstanding bioactivity and biodegradability and exhibits good in vivo biocompatibility [47,52,96]. In one study, Qu et al. prepared negatively charged CS/nano-hydroxyapatite (CS/n-HA) films by the grid-controlled constant-voltage corona charging method for the subsequent osteoinduction studies [97]. The n-HA suspension was dropwise added to the CS solution, and stirred continuously for 2 h, and then dispersed by ultrasound for 0.5 h. Then, the prepared composite material was cross-linked with glutaraldehyde for 2 h, placed on a glass plate, and soaked with NaOH to form a uniform film. The residual glutaraldehyde was removed by repeated rinsing with deionized water and NaBH_4_. Finally, the composite film was charged with a gate control corona for 5 min at room temperature with a corona voltage of −8 kV and a gating voltage of −1 kV. The charge storage capacity was measured by surface potentiometry, and the results showed that the surface charge decreased on the first day and remained stable for the next 9 days. Furthermore, the osteoblasts on the CS/n-HA composite electret film exhibited enhanced adhesion, proliferation, and differentiation abilities compared to the uncharged composite.

In addition, the interstitial position of the porous electret can provide an ideal position to capture charge and increase electrical stability. Wang et al. developed a CS electret porous film with a 3D porous structure using thermally-induced phase separation and a grid-controlled corona charging method [98]. Firstly, CS powder was dissolved in acetic acid, filtered, cross-linked with glutaraldehyde, and then lyophilized at −80 °C. Subsequently, the acidity of the cross-linking solution was neutralized by soaking the porous film with NaOH. After the porous film was separated from the glass dish, it was soaked in NaBH_4_ to remove residual glutaraldehyde, washed repeatedly with distilled water, and freeze-dried. Then, a porous electret film with an interconnected pore structure was obtained. Finally, the porous film was charged by a corona controlled by a charging grid. The results of in vitro experiments showed that the rate of new bone formation under the porous electret film was higher than under the porous yet uncharged film. Moreover, the degradation rate of the porous electret film was also faster.

#### 2.2.7. Collagen and Amino Acid

Collagen is the main structural protein of biological connective tissue and the key component of the extracellular matrix [99]. Moreover, it has good biocompatibility and mechanical properties and has been widely used as a biomedical material [100,101]. Notably, collagen has a dipole group and can be induced into the electret state [102]. To investigate the effect of collagen bio-electrets on cell behavior, Yang et al. prepared negatively charged collagen-coated electrets [102]. Collagen was extracted from pig skin and then dissolved in 0.02 M acetic acid at a concentration of 1.0 mg/mL. The collagen coating was deposited on the bottom of wells of 4-well culture plates by drying collagen solution overnight at room temperature and under UV light. The corona charging method was used to treat collagen coatings to form negative charge deposition on their surfaces. The results showed that collagen bio-electret could regulate the growth of different cell types by changing the intracellular calcium concentration.

Amino acids are an essential building block for sustaining life and vital nutrients for immune cells. It can be involved in protein synthesis as well as many other intracellular metabolisms to support cellular and organismic functions [103,104]. With low elasticity, permittivity, as well as moderate piezoelectric constants, amino acids have emerged as an emerging biological electret material. O’Donnell et al. reported the growth of L-leucine bio-electret crystal films on conductive substrates [105]. Herein, L-leucine was dissolved in deionized water, heated, and stirred until completely dissolved. The solution was then dropped onto a dry copper substrate to produce dense crystalline films. Longitudinal measurements of amino acid films compressed between two copper substrates using a commercial piezoelectric meter showed an average response intensity of +1.57 pC/N for the vertical form and −1.52 pC/N for the reverse form, which indicates the presence of piezoelectric response within the film.

#### 2.2.8. Others

5-fluorouracil (5-FU) is a fluoropyrimidine antimetabolite commonly used in the treatment of various types of malignant tumors [106,107,108,109]. However, as 5-FU has biological toxicity, the local injection may cause side effects such as pain and ulceration at the injection site [110,111]. Therefore, it is necessary to develop a new method to improve the safety of 5-FU. To improve the safety of 5-FU, Yuan et al. developed an electret 5-FU patch to control the drug concentration at the wound site, while reducing the drug concentration in plasma [111]. In this study, a homogeneous solution was obtained by dissolving 5-FU in an ethanol solution containing Eudragit 100 and tributyl citrate. Then, the prepared solution was poured on the PP backing layer to get the desired drug film. Then, the 5-FU transdermal patch was obtained by coating the surface of the adhesive layer with a release pad. Finally, the electret-charged surface was overlaid on the un-adhesive backing layer of the 5-FU patch. The experimental results revealed that the electret electrostatic field would cause a decrease in matrix molecular polarization and adhesion strength, which would change the distribution and interaction of drug molecules and thus increase the drug release from the transdermal film. Moreover, the d_33_ values of the electret 5-FU patch were significantly increased, with 1.13 pC/N and −1.17 pC/N for the −2000 V and 2000 V electret action 5-FU patches, respectively.

## 3. Biomedical Applications

Due to their good performance, high robustness and reliability, long charging life, and ability to provide endogenous electrical stimulation, electrets have attracted widespread interest in a variety of biomedical applications, such as bone regeneration, wound healing, nerve regeneration, drug delivery, wearable electronics, etc. [11,14,23]. In this section, the biomedical applications of electrets were discussed.

### 3.1. Bone Regeneration

**Figure 4:**
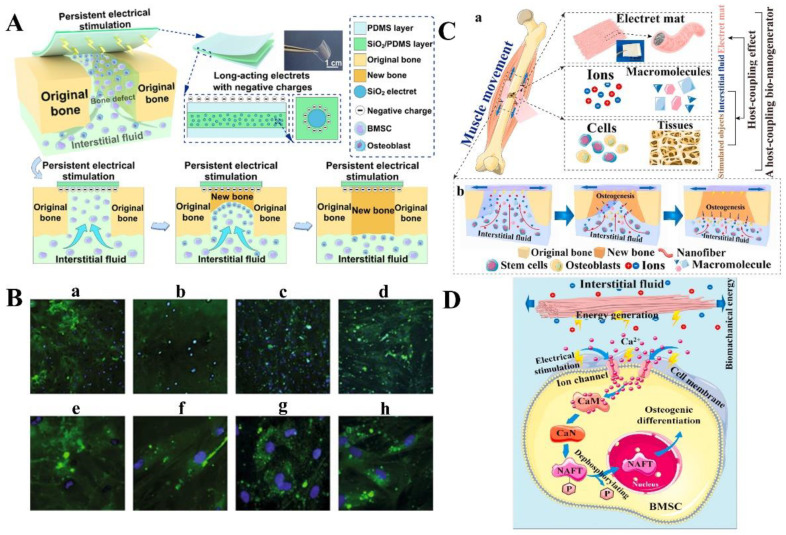
(**A**) Schematic representation of bone defect repair stimulated by continuous electrical stimulation with sandwich-like SiO_2_/PDMS composite film. SiO_2_ nanoparticles were encapsulated and electrically polarized to provide an induced charge and maintain a stable negative potential across the composite film to provide continuous electrical stimulation. Reprinted with permission from Ref. [10]. Copyright 2022, copyright American Chemical Society. (**B**) Detection of osteocalcin in BMMSCs cells cultured for 28 days in the osteogenic differentiation medium (**a**,**e**): without tantalum disc; (**b**,**f**): without electret coating; (**c**,**g**): electret with uniform charge distribution; (**d**,**h**): electret with linear charge distribution; (**a**–**d**) stands for type I collagen; e-h for type II collagen). Reprinted with permission from Ref. [115]. Copyright 2019, copyright Springer Nature. (**C**) Schematic representation of electret HCBG after bone injury in vivo (blue arrow: the direction of muscle movement, black cross: the sutures). Reprinted with permission from Ref. [116]. Copyright 2021, copyright Elsevier. (**D**) A schematic representation of the signaling pathways associated with calcium ions during electrical stimulation of the HCBG. Reprinted with permission from Ref. [116]. Copyright 2021, copyright Elsevier.

As a highly mineralized and metabolically active tissue, bone not only serves as a support that enables humans to walk upright but also provides mechanical protection to organs and is actively involved in regulating blood pH and maintaining mineral homeostasis [112,113]. However, injuries can occur when bones are impacted by strong external forces and some diseases [114]. Therefore, the effective promotion of bone regeneration has been the focus of research. Qiao et al. designed a SiO_2_ electret-doped PDMS (SiO_2_/PDMS) composite electroactive film and investigated its bone regeneration effect, as shown in Figure 4A [10]. The film exhibited good electrical stability over time, and the surface potential could be adjusted to a biopotential that promotes bone growth by regulating the electret concentration. Benefiting from the continuous electrical microenvironment, the composite film promoted the adhesion, proliferation, migration, and osteogenic differentiation of BMMSCs in vitro and significantly promoted the healing of bone defects in vivo. In another study, Qu et al. studied the charge-storage capacity of the negatively charged CS/n-HA films. The cytocompatibility and cellular bioactivity were also studied by seeding rat skull osteoblasts on composite electret films [97]. The results showed that the adhesion, proliferation, and differentiation abilities of osteoblasts on CS/n-HA composite electret film were significantly enhanced. Aleksandrova et al. investigated the ability of osteoarthritis patient-derived BMMSCs to differentiate into osteogenic and chondrogenic lines under the influence of an electret electric field on anodized tantalum surfaces [115]. It was shown that the negatively charged tantalum electret stimulated the proliferation, migration, and metabolic activity of marrow BMMSCs and bone tissue cells. Moreover, the synthesis of protein differentiation markers in BMMSCs was enhanced, and the osteogenic differentiation of BMMSCs was accompanied by significantly enhanced osteocalcin and type I collagen expression (Figure 4B).

In a separate study, Yu et al. designed an electret-based host-coupled biological nanogenerator (HCBG) for electrically stimulated osteogenesis, as schematically illustrated in Figure 4C [116]. After the implantation of porous electret nanofiber mats, the interstitial fluid and the stimulated object were coupled to create a host-coupling effect. The electrical stimulation is capable of increasing cytoplasmic calcium ions to activate osteogenic differentiation. Furthermore, calcium-induced signaling pathways were activated, which significantly promoted the osteogenic differentiation of BMMSCs in vitro and the bone regeneration effect in vivo (Figure 4D). In another study, the porous CS electret films were prepared by Wang et al. for bone regeneration [98]. The in vitro results showed that when porous electret films were cultured with rat osteoblasts, cell proliferation, and differentiation were significantly enhanced compared with porous uncharged films. In addition, in vivo implantation of porous electret films in a rabbit model of calf bone defect showed that the new bone formation rate on porous electret film (feeding duration: 16 weeks) was higher than that under uncharged films.

### 3.2. Wound Healing

**Figure 5:**
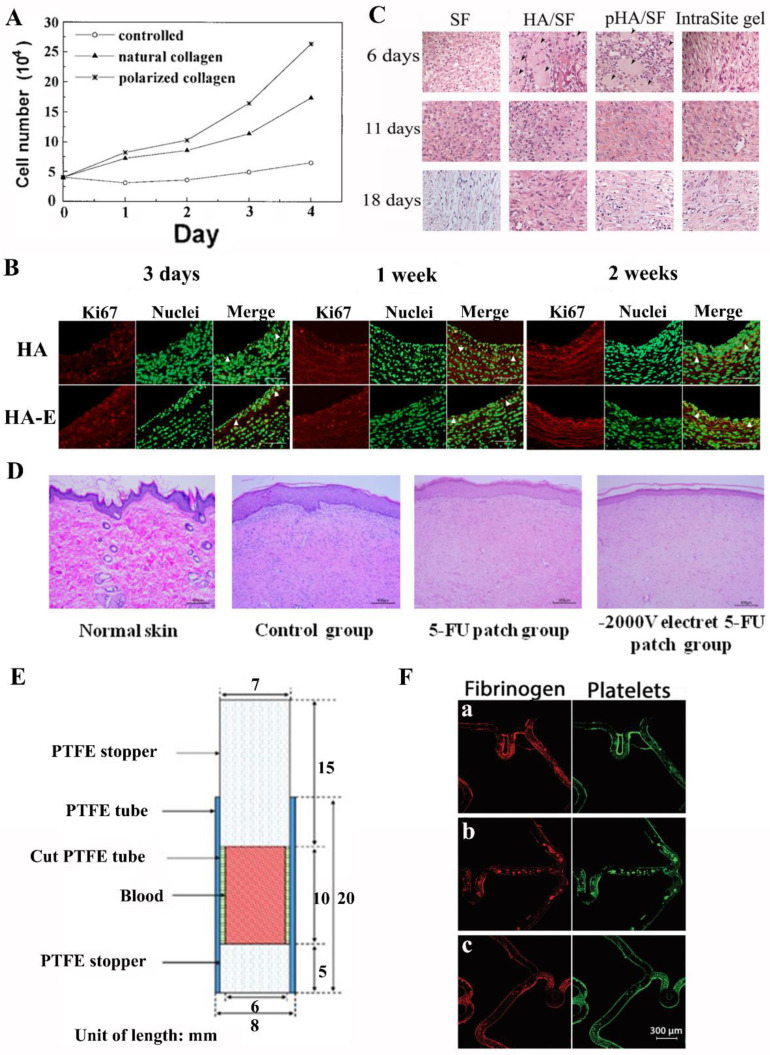
(**A**) The plot of the number of cells grown on different collagen films versus culture time. Reprinted with permission from Ref. [126]. Copyright 1998, copyright John Wiley and Sons, Inc. (**B**) Comparison of representative images of Ki67 immunohistochemistry and the effect of unpolarized HA and HA electret (HA-E) at 3 days, 1 week, and 2 weeks after carotid endothelial resection in rabbits. Reprinted with permission from Ref. [127]. Copyright 2008, copyright Elsevier. Original magnification 200×, scale = 100 μm. (**C**) Histology of dermal wound sections stained with H&E. Wound biopsies from pigs treated with various gels (SF, HA/SF, pHA/SF, and IntraSite gels) were stained for H&E at 6, 11, and 18 days, respectively. Reprinted with permission from Ref. [128]. Copyright 2009, copyright John Wiley and Sons, Inc. Arrows indicate intruding inorganic particles. (**D**) Histological image of skin tissue stained with H&E on day 28. Reprinted with permission from Ref. [110]. Copyright 2021, copyright Elsevier. (**E**) Schematic representation of the cross-section of a tube with blood sample. Reprinted with permission from Ref. [131]. Copyright 2019, copyright Springer Nature. (**F**) Representative confocal microscopic images of (**a**) BMS, (**b**) DES, and (**c**) e−DES. Fibrinogen deposition is represented on the left and platelet deposition is represented on the right. Reprinted with permission from Ref. [55]. Copyright 2022, copyright AIP Publishing.

Wounds can result from trauma, vascular dysfunction, diabetes, hypertension, and rheumatic and inflammatory disease [117,118,119,120,121]. Wound healing is a complex process designed to restore damaged skin in order to maintain a state of tissue homeostasis [122,123]. Currently, it has been reported that the wound-healing process is able to be accelerated by simulating the natural current of injury, which has become a new research hotspot in the field of wound healing [124,125]. Sun et al. prepared a collagen-based biological electret material and investigated its potential in promoting wound healing [126]. The results showed that polarized collagen electret had a significant effect on cell growth in vitro (Figure 5A). In addition, Nagai et al. investigated whether electrically polarized HA could alter re-endothelialization and intimal hyperplasia after endothelial resection in rabbit carotid arteries [127]. HA electrets and unpolarized HA were used as controls and mixed with agarose gels to make powders and applied to the external surface of the carotid artery. As shown in Figure 5B, histological analysis showed that the number of Ki67 positive cells in the inner film of the HA electret group was significantly reduced, while the number of CD31 positive cells in the lumen surface was significantly higher than that in the control group. This study successfully demonstrated that HA electret could significantly inhibit intimal hyperplasia after endothelial resection by accelerating endothelial cell regeneration and enhancing the apoptosis of medial smooth muscle cells. In another study, polarized HA powder (pHA) was added to silk fibroin protein (SF) and gelatinized the composite gel. Then, the composite gel was applied to full-thickness pig skin wounds to observe the healing effect [128]. The results proved that the pHA/SF gel could effectively promote the maturation of fibroblasts and facilitate the epithelial cell and matrix formation (Figure 5C). Yuan et al. prepared 5-FU patches, as well as PP electret 5-FU patches (charged with a −1000 V or −2000 V electric field), and applied them to rats hypertrophic scar wounds [110]. The inhibition effect of negative electrets on hypertrophic scar growth was investigated by hematoxylin and eosin (H&E) staining, and the results are shown in Figure 5D. The results displayed that a negative 5-FU patch electret enhanced the penetration and retention of 5-FU in scarred skin, and the inhibition effect was better than a single 5-FU patch (experimental duration: 28 days).

Moreover, electrets have been proven to be effective in antithrombotic therapy. The most serious complication of percutaneous coronary intervention with stents is stent thrombosis (ST), which obstructs blood removal and leads to treatment interruption [129,130]. Using the triboelectric charging method, Ogino et al. proposed a PTFE electret tube to prevent the coagulation of intravascular indwelling catheters [131]. By rubbing the inner wall using a round glass rod, an electrostatic charge was produced inside the PTFE tube. Figure 5E shows a schematic cross-section of the tube with the sealed blood, and no detectable thrombin adhesion in the inner wall was observed using a microscope. In another study, Zafar et al. found that electret coating technology could generate lasting negative charges on the surface of the stent, which makes the stent inherently anti-thrombotic [55]. Bare-metal stents (BMS), standard uncoated DES (DES), and electret-coated DES (e-DES) were exposed to arterial blood flow conditions using a ring perfusion chamber. Immunofluorescence staining and confocal microscopy were used to analyze the deposition of fibrinogen and platelets on the stent surface. As shown in Figure 5F, the stent surface fibrinogen and platelet coverage area followed the sequence of e-DES < DES < BMS and the size of fibrinogen deposition did not differ between stents, implying that the electret coating provided significant antithrombotic properties for DES stents.

### 3.3. Nerve Regeneration

Nerve injuries are usually caused by accidents, tumor removal, and compression syndromes, and may lead to extreme numbness, neuropathic pain, and decreased or even loss of muscle strength [132,133,134,135]. Moreover, motor and sensory recovery after nerve injury is limited by the rate of peripheral nerve regeneration and the decreased regenerative potential of muscle nerves after prolonged atrophy [136]. As a result, designing an artificial system to promote nerve regeneration and repair is essential. Recently, Miura et al. used an electret PTFE tube and an electrically neutral PTFE tube as nerve guide channels to repair the 5 mm nerve gap in the mouse sciatic nerve [137]. Four weeks after the implantation, PTFE with positive or negative charges showed nerves with more myelin axons than the uncharged ones, which indicated that the electrified nerve guide channel can enhance the regeneration of the peripheral nerve. In addition, they demonstrated that positively charged coating materials such as polylysine are able to improve the attachment of neurons in vitro [138]. In another study, mouse neuroblastoma (Nb2a) cells were cultured in serum-containing and serum-free media with positive, negative, and uncharged FEP substrates. The experimental results showed that there were no differences in surface chemistry and morphology and no significant differences in cell attachment level among these FEPs. However, under both medium conditions, the positive substrates showed significantly higher levels of neurite growth than the negative and uncharged substrates, while the greatest growth level was observed for substrates charged to +1000 V.

**Figure 6:**
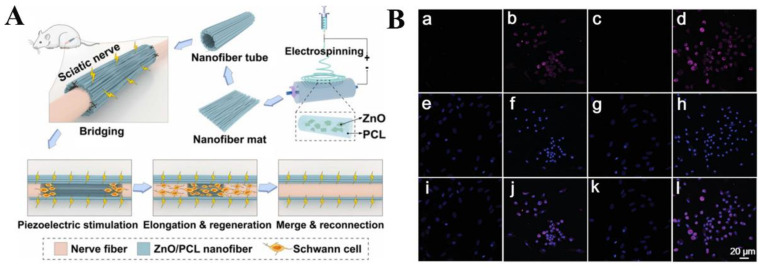
(**A**) Schematic design of electrospun composite nanofibers for rapid regeneration of peripheral nerves. Electrospun PCL was wrapped in a 0.5 mm diameter nanofiber tube with a ZnO nanofiber mat and then implanted into PZNF for nerve bridging. PZNF achieves recruitment of Schwann cells, regeneration of injured axons, and finally reconnection of axons through piezoelectric stimulation. Reprinted with permission from Ref. [139]. Copyright 2022, copyright Elsevier. (**B**) Immunofluorescence images of Ki67 in different samples of NSCs. NSCs were cultured on (**a**,**e**,**i**) TCP, (**b**,**f**,**j**) PLLA nanofiber film, (**c**,**g**,**k**) TCP, and (**d**,**h**,**l**) PLLA nanofiber film treated with 300 W ultrasound, respectively. (**i**–**l**) Merged image of Ki67 and matching. Reprinted with permission from Ref. [140]. Copyright 2022, copyright John Wiley &and Sons, Inc.

Since then, ever-increasing strategies to promote nerve regeneration have been widely developed. Mao et al. developed piezoelectric nerve conduits by electrospinning composite PCL/ZnO nanofibers (PZNF) [139]. As illustrated in Figure 6A, the material was able to continuously generate piezoelectricity and provide stable endogenous electrical stimulation, which significantly increased the expression of nerve growth factor/vascular endothelial growth factor and significantly decreased the time for sciatic nerve repair (within 4 weeks). Lu et al. also reported a simple method to promote the expansion and maintain stemness of neural stem cells (NSC) using piezoelectric nanomaterial-triggered radio stimulation [140]. The good piezoelectric properties of poly-L-lactic acid (PLLA) nanofiber film, which was prepared by electrospinning, are beneficial to the release of electrons after conversion. Through immunofluorescence staining assay (Figure 6B), it was confirmed that these electrical signals significantly enhanced the proliferation of NSCs, and that the neural differentiation ability and function of NSCs were not weakened during the proliferation process.

In another study, Jin et al. developed a wearable neuroelectric stimulation system with physiological biofeedback, self-regulation, and self-powered functions [141]. Using vagal impulse-controlled respiratory movements as a biomechanical source, signals physiologically related to the overall vagal impulse peak envelope are synchronized and generated. Simultaneously dissipates excessive charge accumulation and allows for persistent electrical stimulation activity in nascent nerve cells and tissues that can regenerate relevant signaling pathways.

### 3.4. Drug Delivery

**Figure 7:**
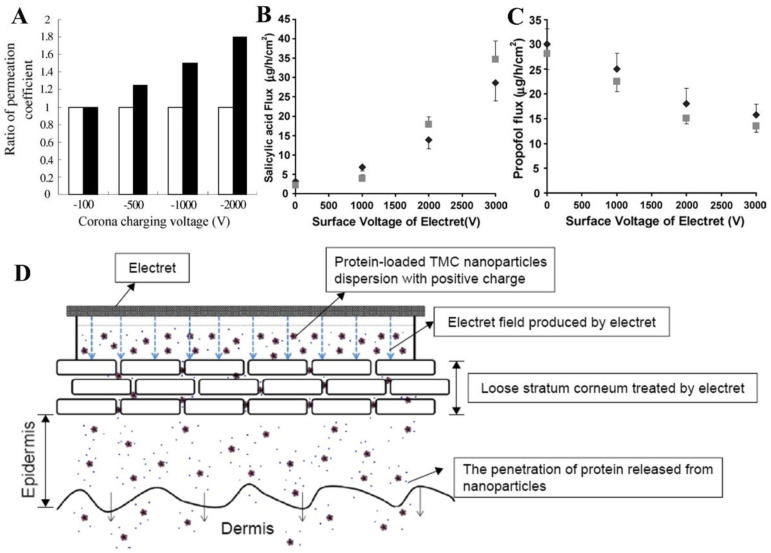
(**A**) Effect of corona−charged electret (black) and control group (white) on in vitro transdermal enhancement. Reprinted with permission from Ref. [148]. Copyright 2001, copyright Elsevier. (**B**) Salicylic acid transport flux through the epidermis of positively charged electrets (gray) and negatively charged electrets (black) at different surface voltages. Reprinted with permission from Ref. [150]. Copyright 2008, copyright Pharmaceutical Society of Japan. (**C**) Propofol transports flux through the epidermis of positively charged electrets (gray) and negatively charged electrets (black) at different surface voltages. Reprinted with permission from Ref. [150]. Copyright 2008, copyright Pharmaceutical Society of Japan. (**D**) Schematic representation of the transdermal delivery system with TMC NPs bound to electrets. Reprinted with permission from Ref. [151]. Copyright 2016, copyright Dove Medical Press Ltd.

Traditional drug therapy has some disadvantages, such as non-targeting, short duration of drug action, poor solubility of drug molecules, and side effects [142,143]. Therefore, local drug delivery may be an effective way to reduce side effects and improve therapeutic efficacy. On this basis, targeted drug delivery and controlled release drug delivery systems (DDS) have attracted extensive attention in the biomedical field [144,145]. Electrical stimulation is an attractive method to spatiotemporally modulate the on-demand release of drugs [146,147]. Cui et al. investigated the promoting effect of electrets on in vitro transdermal drug delivery [148]. Modified Franz diffusion cells were used in the experiment in which rat skin was placed between two half cells, and the electret film of the model compound was overlaid on the donor cells. The received solution was extracted at a predetermined time and the drug (methyl salicylate) concentration was determined by high-performance liquid chromatography (HPLC). The results showed that the penetration flux of the electret group was significantly better than that of the control group (Figure 7A). This suggests that the microcurrent and electrostatic field generated by the electret will affect the transdermal effect of the drug. Subsequently, they charged the Al electrodes coated PP films to prepare electrets (operating voltage: −500 V, −1000 V, and −2000 V) and generate electric fields [149]. The model drug cyclosporine A (CsA) was loaded on the patch, and the release behavior was studied in vitro. The results showed that the electrostatic field generated by the electret could induce the polarization of the drug in the patch as well as enhance its release in a charging voltage manner. After that, they developed a 5-FU electret patch and demonstrated that the electrostatic field of the electret could cause a decrease in the polarization and cohesion of matrix molecules, alter the distribution and interaction of drug molecules within the patch, and thus increase the amount of drug released [111].

In another study, the effect of electret exposure on skin permeability was explored [150]. Salicylic acid and propofol were used as test diffusers. The results showed that the electret enhanced the permeability of the pig epidermis to salicylic acid, and the enhancement factor increased with the increase of the surface voltage but was irrelevant to the nature of the charge (Figure 7B). Meanwhile, the electret reduced the skin’s permeability to the lipophilic diffuser propofol (Figure 7C). It is most likely that the electret exposure makes the lipid domains of the cuticle more permeable to polar molecules, which in turn impedes the diffusion of nonpolar diffusers. Tu et al. developed protein-loaded N-trimethyl chitosan nanoparticles (TMC NPs) using the ion-gel method, to investigate whether the transdermal delivery of protein drugs by TMC NPs could be enhanced by PP electrets, which were charged using a corona charging system [151]. The mechanism is shown in Figure 7D. Corresponding experiments including in vitro skin penetration assays and anti-inflammatory effects were performed, which showed that transdermal delivery of protein drugs in TMC NPs was considerably improved in the presence of electrets. In addition, Li et al. successfully prepared a flexible, biocompatible drug-laden composite electret (nCW) using thermal polarization [152]. nCW’s nano effects increased the charge density and the “locked” dipole structure of the polymer matrix improved the charge stability. This design of locked dipole particles based on a dual-network polymer matrix opens up new ideas for the construction of polymer-based bioelectretes.

### 3.5. Wearable Electronics

**Figure 8:**
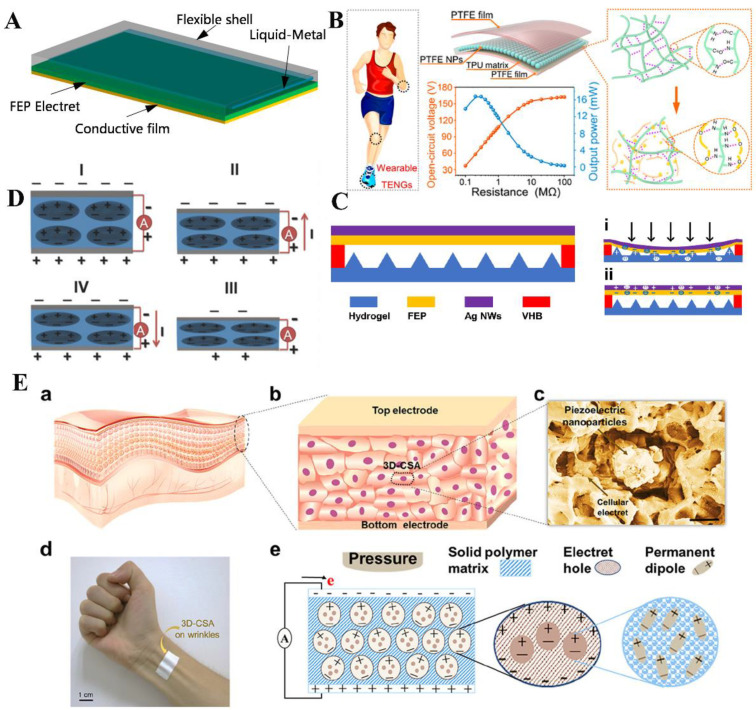
(**A**) Schematic diagram of flexible stretchable energy harvester based on liquid metal and FEP electret films. Reprinted with permission from Ref. [158]. Copyright 2020, copyright Multidisciplinary Digital Publishing Institute. (**B**) Schematic diagram of the structure of TENG and the acquisition of biomechanical energy from various parts of the body. Reprinted with permission from Ref. [159]. Copyright 2021, copyright American Chemical Society. (**C**) Schematic diagram of the structure of EHTS and EHTS in the compressed (**i**) and released (**ii**) states. Reprinted with permission from Ref. [160]. Copyright 2021, copyright Multidisciplinary Digital Publishing Institute. (**D**) Illustration of working principle of electret flexible generator in (**I**) initial state, (**II**) extrusion state, (**III**) equilibrium state, and (**IV**) release state, respectively. Reprinted with permission from Ref. [161]. Copyright 2015, copyright John Wiley and Sons, Inc. (**E**) Structure and working principles of 3D-CSA: (**a**) schematic diagram of human epidermis structure, (**b**) 3D-CSA diagram simulating the structure of the human epidermis, (**c**) SEM image of cell sensor unit structure, (**d**) photograph of 3D-CSA, (**e**) schematic diagram of the working principles of 3D-CSA. Reprinted with permission from Ref. [162]. Copyright 2018, copyright American Chemical Society.

At present, wearable medical devices have been widely used in clinical diagnosis and health monitoring, including body fluid detection [153], neurological disease detection [154], cardiovascular monitoring [155], sleep monitoring [156], and respiratory monitoring [157]. The electret is one of the most promising platforms for the construction of high-performance self-powered wearable electronic products due to its high flexibility, light weight, large charge storage, biocompatibility, and good piezoelectric properties [11,23]. Based on liquid metal and FEP electret films, Xie et al. designed a flexible stretchable energy harvester for wearable devices [158]. The structure diagram is shown in Figure 8A. The liquid metal of gallium was used to form stretchable electrodes and give the induction energy harvester good stretchability and flexibility. The solid electrode was wrapped in a silicone rubber shell and then combined with the conductive film and FEP electret film to fabricate an energy harvester. The fabricated energy harvester was mounted on a human elbow to collect the energy generated by elbow bending. The experimental results showed that the flexible stretchable energy harvester was able to adapt to elbow bending well as well as convert elbow movement into electrical energy, which can be used for lighting LED of wearable watches. In another work, Wang et al. proposed a material optimization strategy to improve the performance of triboelectric nanogenerators (TENG) while reducing the matching impedance [159]. They used an electret composite film with an adjustable dielectric constant (thermoplastic polyurethane (TPU) matrix containing polyethylene glycol (PEG) and PTFE nanoparticles) as the triboelectric layer. The structure is shown in Figure 8B. The internal capacitance and injected charge density of the TENG were significantly increased by optimizing the dielectric permittivity of the composite, resulting in higher output power and lower impedance. Chen et al. prepared a double-mesh ionic hydrogel film by solution displacement treatment [160]. The hydrogel film can act as a flexible electrode and triboelectric layer. An electret/hydrogel-based tactile sensor (EHTS) was designed and fabricated by combining it with FEP film with corona (Figure 8C). With a mixture of triboelectric and electrostatic effects, the output performance of the device was significantly improved to withstand temperatures in a wide range of −10 °C to about 40 °C and could be further integrated into masks for human respiratory monitoring.

Notably, 3D porous materials are also widely used in wearable devices and energy harvesters due to their high porosity and interconnected pore structures. Wu et al. constructed an efficient human energy harvesting and self-powered human health monitoring system by using cellular PP electrets with excellent physical and electrical properties [161]. The maximum peak power density of the electret flexible generator is 52.8 mW/m^2^. In wearable and health monitoring applications, the self-powered human bio-signal detection sensor has been demonstrated to sensitively detect cough movements and arterial beats, as shown in Figure 8D. This study showed that the system has good compatibility and potential for application in human health monitoring, which opens a new avenue for the development of portable and wearable electronic systems. In another study, Yan et al. proposed a flexible 3D cellular sensor array (CSA) [162]. The CSA is composed of 3D cell electret and caged piezoelectric nanoparticles, which have the advantages of ultra-thin (80 μm), ultra-lightweight, and having high mechanical durability. The structure design and working principle are shown in Figure 8E. Due to its rigid and symmetrical structure, 3D-CSA is capable of performing the same operation from both sides and has been successfully used to measure human heartbeats and monitor sleep quality.

## 4. Summary and Perspectives

A large number of studies have shown that electrets have the advantages of permanent polarization, low cost, stable performance, and good biocompatibility. More importantly, the ability to generate endogenous electrical stimulation under the action of an external electric field has greatly contributed to its rapid development in the biomedical field. In this review, we systematically summarized the recent development, classification, and fabrication methods of electret materials and further introduced some representative advances of electrets in the biomedical fields. Their applications cover most areas of biomedicine, such as bone regeneration, wound healing, nerve regeneration, drug delivery, and wearable electronics. Although considerable progress and promising achievements have been made in this direction, some key issues remain to be considered in the future in order to further advance the development of electrets. Here is a summary of the main challenges.

(1) Simplifying the charging method

Charging or polarization is the most representative characteristic of a dielectric. To date, different charging methods have been developed, such as corona charging, soft X-ray irradiation, thermal charging, contact charging, and electron beam injection. Nevertheless, these methods of charging involve factors that limit industrial-scale production. Consequently, there would be great potential to simplify the charging process.

(2) Enhancing the charge density

Charge density is commonly used to assess the charge storage capacity of electrets. Most of the existing electret charges are stored on the surface of dielectric materials and often do not have sufficient charge density, which is far from the requirements of practical applications. Thus, in terms of enhancing the charge density, a possible future development direction is to promote the generation of charge inside the dielectric material, such as through the fabrication of solid porous electrets or hydrogel electrets with the network structure.

(3) Developing degradable platforms

Notably, many electrets do not degrade easily, especially polymer electrets, which could cause environmental pollution. Thus, there is an urgent need to develop biodegradable electret materials. The use of natural biomaterials (such as amino acids and proteins), with proven charge storage properties, will be a promising direction in the future.

## Figures and Tables

**Table 1:** Classification, fabrication method, and piezoelectric coefficient of common electret.

Classification	Material Name	Fabrication Method	Piezoelectric Coefficient d_33_/d_31_ (pC/N)	Ref.
Inorganic	Silicon dioxide (SiO_2_)	High-temperature sintering	4	[53]
	Hydroxyapatite (HA)	Slip casting	6.8	[54]
	Tantalum pentoxide (Ta_2_O_5_)	Vacuum plasma spray technique	-	[55]
	Titanium dioxide (TiO_2_)	Solution cast and mechanical rolling	41	[56]
Macromolecules	Polyethylene (PE)	Extrusion film blowing	935	[57]
	Polypropylene (PP)	Crystallization self-reinforcement foaming	700	[58]
	Polytetrafluoroethylene (PTFE)	Secret commercial technology	1300	[59]
	Polyvinylidene fluoride (PVDF)	Freeze casting	264	[60]
	Fluorinated ethylene propylene (FEP)	Template	1000	[61]
	Chitosan (CS)	Solvent casting	11.29	[62]
	Amino acid	Evaporative crystallization	0.9	[63]

## Data Availability

Not applicable.
